# Supplementary material for: Bidirectional genome-wide CRISPR screens reveal host factors regulating SARS-CoV-2, MERS-CoV and seasonal HCoVs
Source: Res Sq. 2021 May 27:rs.3.rs-555275. Preprint. [Version 1] doi: 10.21203/rs.3.rs-555275/v1 (PMC8168385; doi:10.21203/rs.3.rs-555275/v1)
Supplement: Supplement 6 — Fig. S1. a. Clustermap showing correlations of log-fold change values relative to plasmid DNA across replicates in the Vero E6 screen from the present study. Population 1 (Pop 1) and Population 2 (Pop 2) refers to 2 independent library transductions, in which screens 1A, 1B and 2 refer to biological replicates of SARS-CoV-2 infection in Pop 1 and screen 2 refers to one biological replicate of SARS-CoV-2 infection in Pop 2. “Initial” refers to the uninfected condition. b. Volcano plot showing the top genes conferring resistance (right, blue) and sensitivity (left, red) to SARS-CoV-2 when knocked out in Vero E6 cells for this screen and the screen conducted by Wei et al. 2021 (Wilen; 27). The gene-level z-score and -log10(FDR) were calculated after averaging across conditions (of note, the FDR value for ACE2 is effectively zero but has been assigned a -log(FDR) value for plotting purposes). c. Comparison between genome-wide screens conducted in A549 cells overexpressing ACE2 by Daniloski et al. (Sanjana; 24) and Zhu et al. (Zhang; 28) using the GeCKOv2 and Brunello libraries, respectively. d. Pair-wise comparison between genome-wide screens conducted in Huh7.5.1-ACE2-TMPRSS2, Huh7.5, and Huh7 cells by Wang et al. (Puschnik; 26), Schneider et al. (Poirier; 25), and Baggen et al. (Daelemans; 23), respectively, using the GeCKOv2 and Brunello libraries as indicated. Annotated genes include top 3 resistance hits from each screen as well as genes that scored in multiple cell lines based on the criteria used to construct the Venn diagram in Fig. 1d. Fig. S2. a. Calu-3 cells stably expressing Cas9 were transduced with a lentiviral vector expressing the puromycin resistance gene and GFP, as well as a sgRNA targeting the GFP coding sequence (XPR_047). The percentage of puromycin-resistant cells which did not express detectable levels of GFP was scored by flow cytometry 8–10 days post-transduction. b. and c. Calu-3 cells stably expressing dCas9-VP64 were transduced or not with l [file NIHPPRS555275v1-supplement-6.pdf]

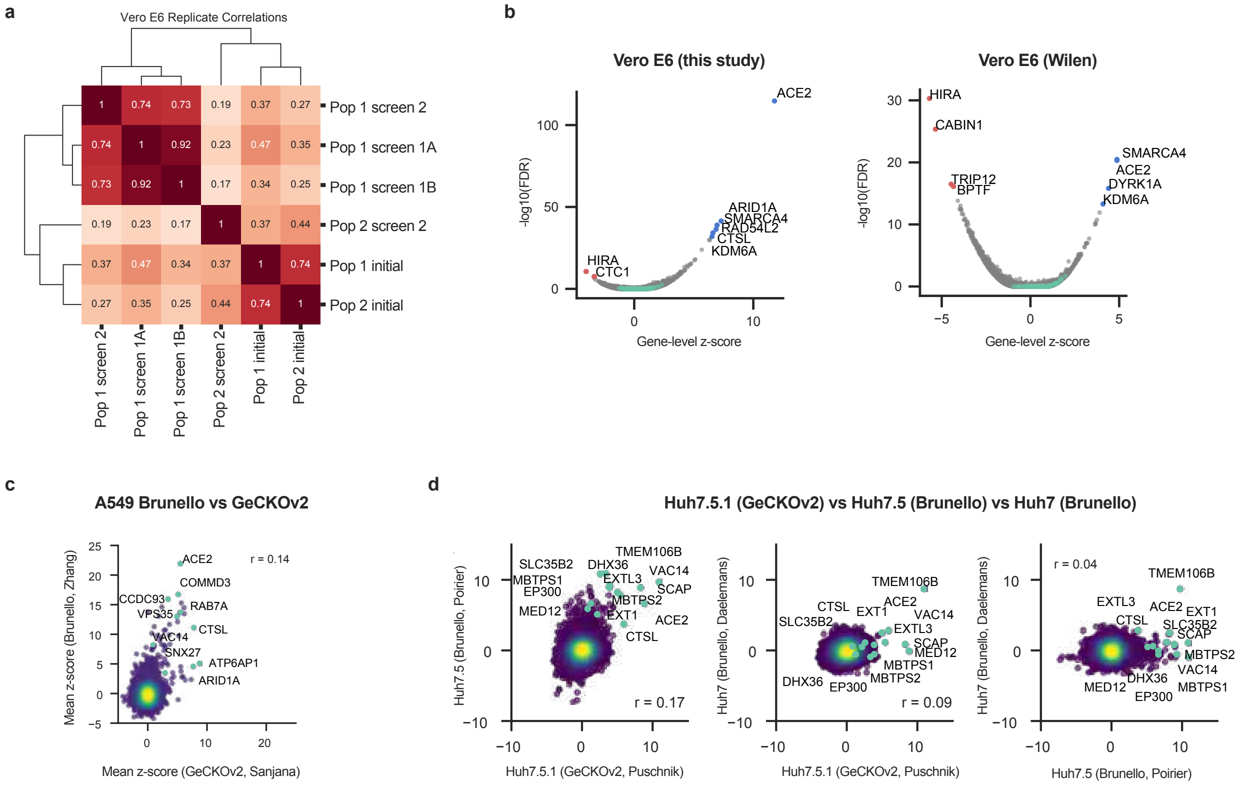

Fig. S1.
a. Clustermap showing correlations of log-fold change values relative to plasmid DNA across replicates in the Vero E6 screen from the present study. Population 1 (Pop 1) and Population 2 (Pop 2) refers to 2 independent library transductions, in which screens 1A, 1B and 2 refer to biological replicates of SARS-CoV-2 infection in Pop 1 and screen 2 refers to one biological replicate of SARS-CoV-2 infection in Pop 2. "Initial" refers to the uninfected condition.
b. Volcano plot showing the top genes conferring resistance (right, blue) and sensitivity (left, red) to SARS-CoV-2 when knocked out in Vero E6 cells for this screen and the screen conducted by Wei et al. 2021 (Wilen; [27]). The gene-level z-score and -log10(FDR) were calculated after averaging across conditions (of note, the FDR value for ACE2 is effectively zero but has been assigned a -log(FDR) value for plotting purposes).
c. Comparison between genome-wide screens conducted in A549 cells overexpressing ACE2 by Daniloski et al. (Sanjana; [24]) and Zhu et al. (Zhang; [28]) using the GeCKOv2 and Brunello libraries, respectively.
d. Pair-wise comparison between genome-wide screens conducted in Huh7.5.1-ACE2-TMPRSS2, Huh7.5, and Huh7 cells by Wang et al. (Puschnik; [26]), Schneider et al. (Poirier; [25]), and Baggen et al. (Daelemans; [23]), respectively, using the GeCKOv2 and Brunello libraries as indicated. Annotated genes include top 3 resistance hits from each screen as well as genes that scored in multiple cell lines based on the criteria used to construct the Venn diagram in **Fig. 1d**.

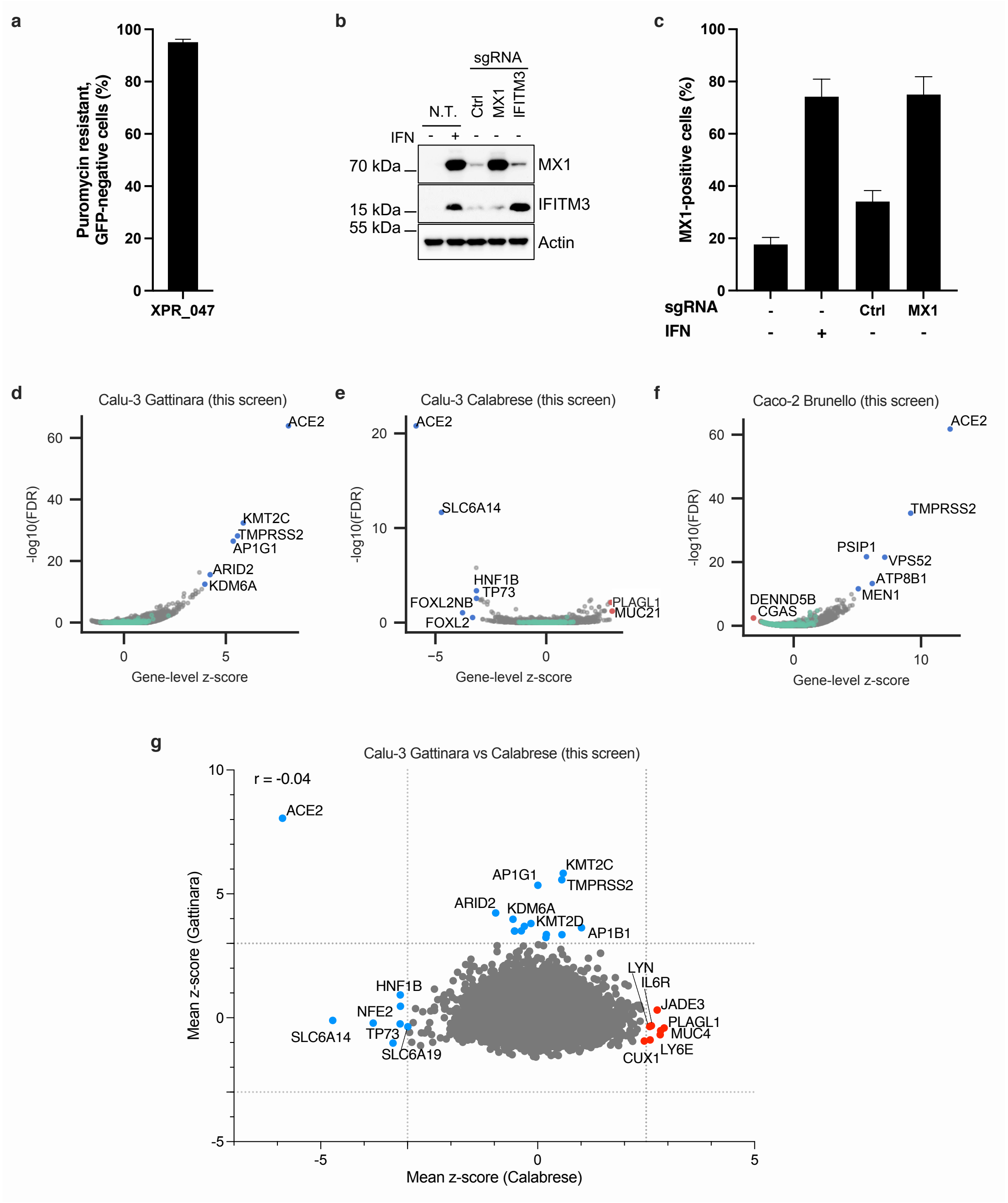

Fig. S2.

a. Calu-3 cells stably expressing Cas9 were transduced with a lentiviral vector expressing the puromycin resistance gene and GFP, as well as a sgRNA targeting the GFP coding sequence (XPR_047). The percentage of puromycin-resistant cells which did not express detectable levels of GFP was scored by flow cytometry 8-10 days post-transduction.

b. and **c.** Calu-3 cells stably expressing dCas9-VP64 were transduced or not with lentiviral vectors expressing sgRNAs targeting either nothing (Ctrl), *MX1* or *IFITM3* promoter and puromycin-selected for 8-10 days. In parallel, non-transduced (N.T.) cells were treated or not with 1000 U/mL interferon for 24h. Cells were harvested for immunoblot analysis (**b**) or fixed, permeabilized and stained with an anti-MX1 antibody and an Alexa Fluor 488 secondary antibody and analyzed by flow cytometry (**C**). Biological duplicates (**a, c**) and a representative immunoblot (**b**) are shown.

d. Volcano plot showing the top genes conferring resistance (right, blue) to SARS-CoV-2 when knocked out in Calu-3 cells. This screen did not have any sensitization hits. The gene-level z-score and -log10(FDR) were calculated after averaging across replicates.
e. Volcano plot showing the top genes conferring resistance (right, red) and sensitivity (left, blue) to SARS-CoV-2 when overexpressed in Calu-3 cells. The gene-level z-score and -log10(FDR) were calculated after averaging across replicates.
f. Volcano plot showing the top genes conferring resistance (right, blue) and sensitivity (left, red) to SARS-CoV-2 when knocked out in Caco-2 cells. The gene-level z-score and -log10(FDR) were calculated after averaging across replicates.
g. Comparison between gene hits in Calu-3 KO and activation screens. Dotted lines indicated mean z-scores of -3 and 2.5 or 3 for each screen. Proviral and antiviral genes are indicated in blue and red, respectively.

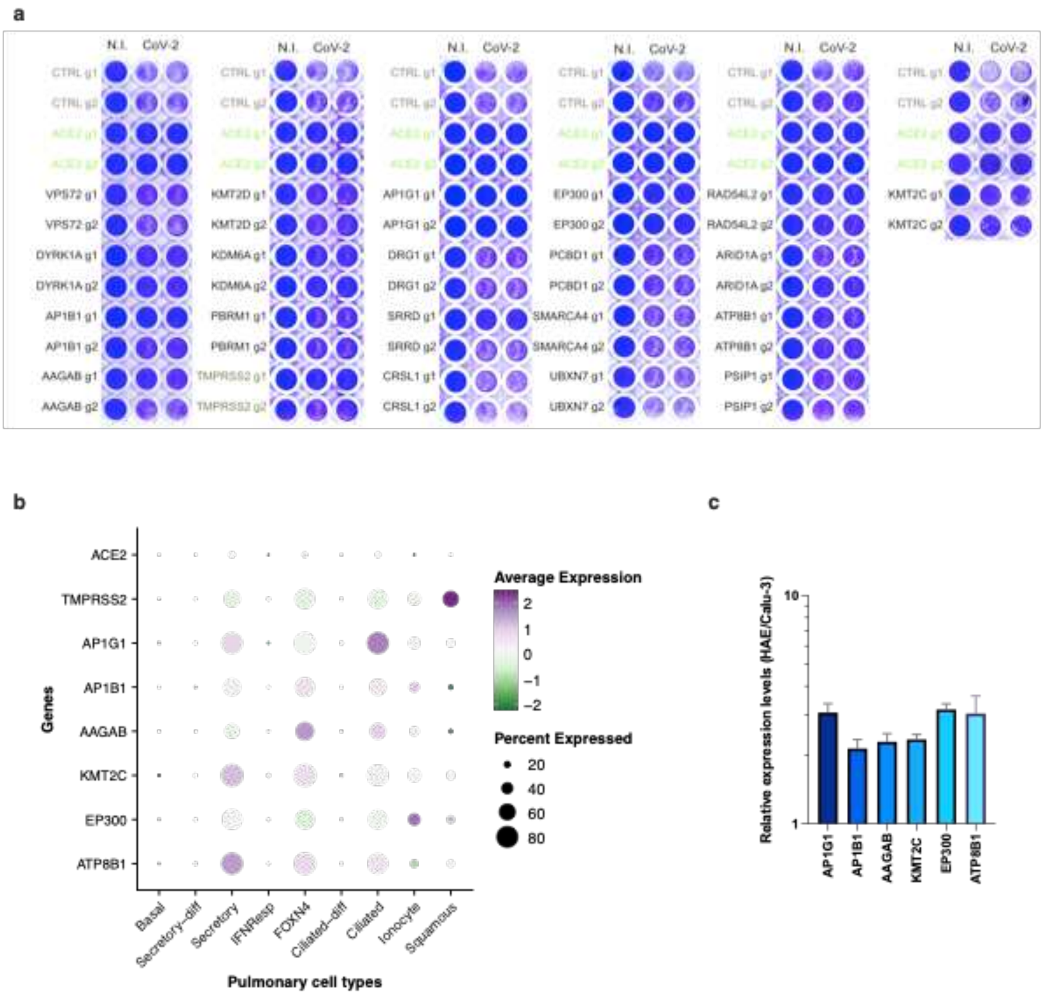

Fig. S3.

a. SARS-CoV-2 induced cytopathic effects in candidate KO cell lines.

Calu-3-Cas9 cells were stably transduced to express 2 different sgRNAs (g1, g2) per indicated gene and selected for 10-15 days. Cells were infected by SARS-CoV-2 at MOI 0.005 and ~5 days later stained with crystal violet. Representative images are shown.

b. Dot plot depicting the expression levels of the best validated genes in the different cell types from the respiratory epithelium, from Chua et al. data set [40]. Expression levels in COVID-19 versus healthy patients are color coded; the percentage of cells expressing the respective gene is size coded, as indicated.

c. Relative expression levels of the identified dependency factors in primary human airway epithelial (HAE) cells compared to Calu-3 cells. RNA samples from 3 independent experiments (and 3 independent donors for HAE cells), described in [30], were analyzed by RT-qPCR using the indicated taqmans.

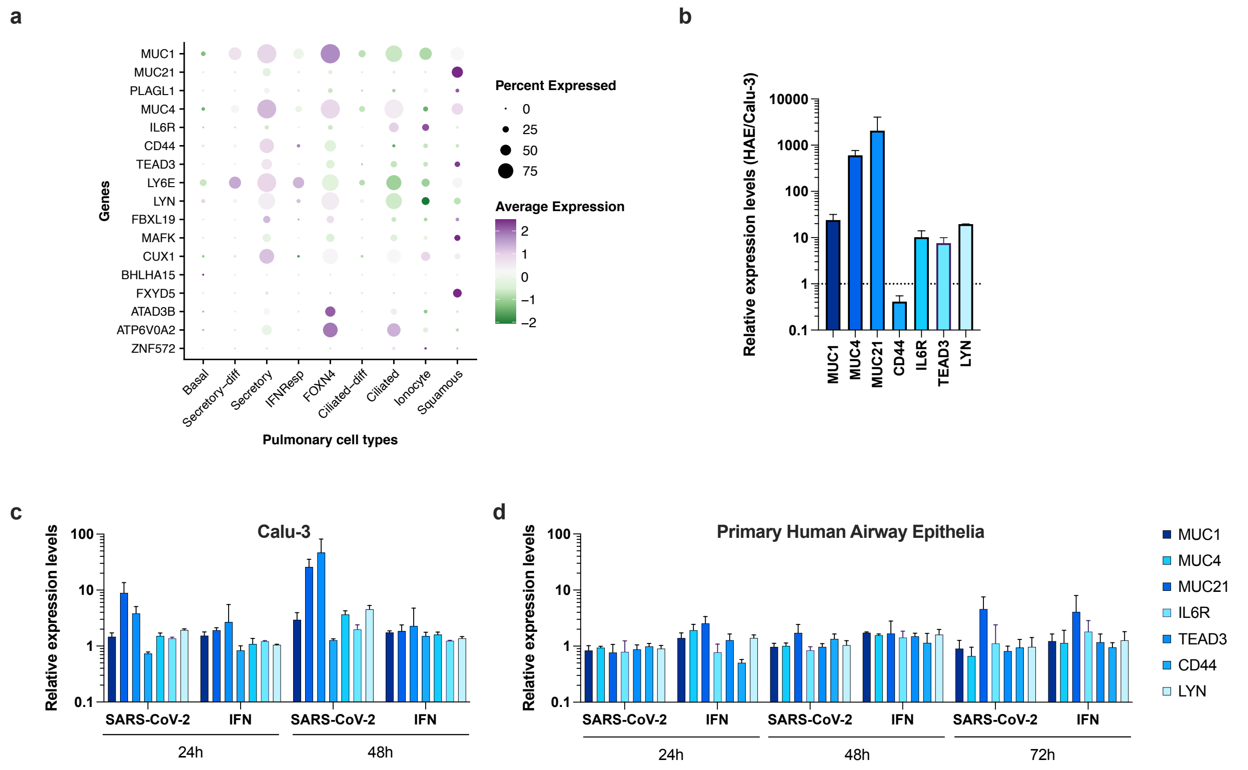

Fig. S4.

a. Dot plot depicting the expression levels of the best validated antiviral genes in the different cell types from the respiratory epithelium, from Chua et al. data set [40]. Expression levels in COVID-19 versus healthy patients are color coded; the percentage of cells expressing the respective gene is size coded, as indicated.

b. Relative expression levels of a selection of the antiviral factors in primary human airway epithelial cells (HAE) compared to Calu-3 cells. RNA samples from 3 independent experiments, described in [30], were analyzed by RT-qPCR using the indicated taqmans.

c-d. Impact of SARS-CoV-2 infection and interferon treatment on antiviral factor expression in HAE (**c**) **and Calu-3 cells** (**d**), as indicated, in samples from 3 independent experiments from [30].

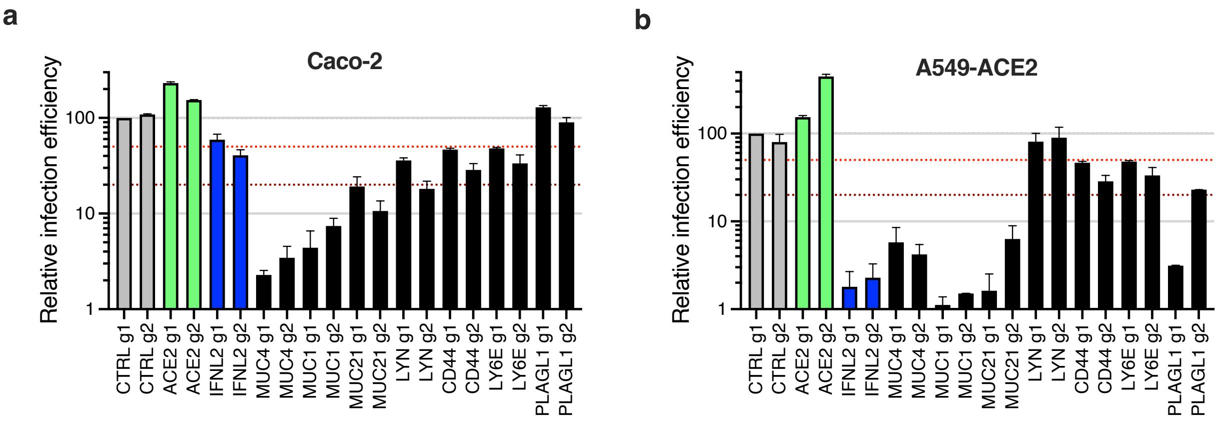

Fig. S5. Impact of the identified antiviral genes on SARS-CoV-2 in Caco-2 and A549-ACE2 cells. Caco-2-dCas9-VP64 (**a**) and A549-ACE2-dCas9-VP64 (**b**) cells were stably transduced to express 2 different sgRNAs (g1, g2) per indicated gene promoter, or negative controls (CTRL) and selected for at least 10-15 days prior to SARS-CoV-2 mNG infection. The percentage of infected cells was scored 48h later by flow cytometry. Relative infection efficiencies (mean and SEM) are shown for 2 independent experiments. The red and dark red dashed lines represent 50% and 80% inhibition, respectively.

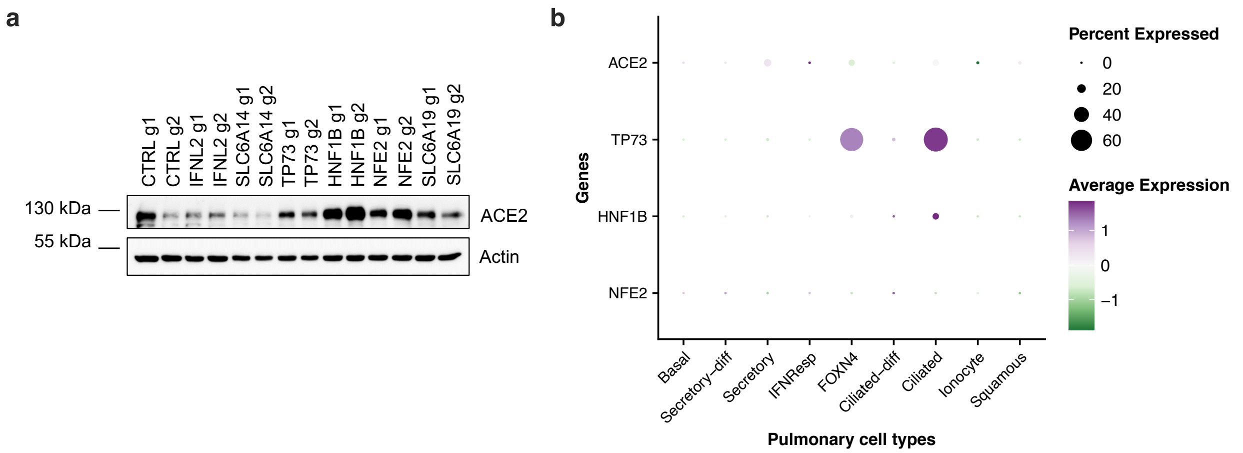

Fig. S6.

a. ACE2 expression in CRISPRa cell lines.

Calu-3-Cas9 cells were stably transduced to express 2 different sgRNAs (g1, g2) per indicated gene and selected for 10-15 days (parallel samples from **Fig. 7- 8**). The cells were lysed and expression levels of ACE2 were analyzed, Actin served as a loading control. A representative immunoblot is shown.

b. Dot plot depicting the expression levels of the best validated proviral genes in the different cell types from the respiratory epithelium, from Chua et al. data set [40]. Expression levels in COVID-19 versus healthy patients are color coded; the percentage of cells expressing the respective gene is size coded, as indicated.

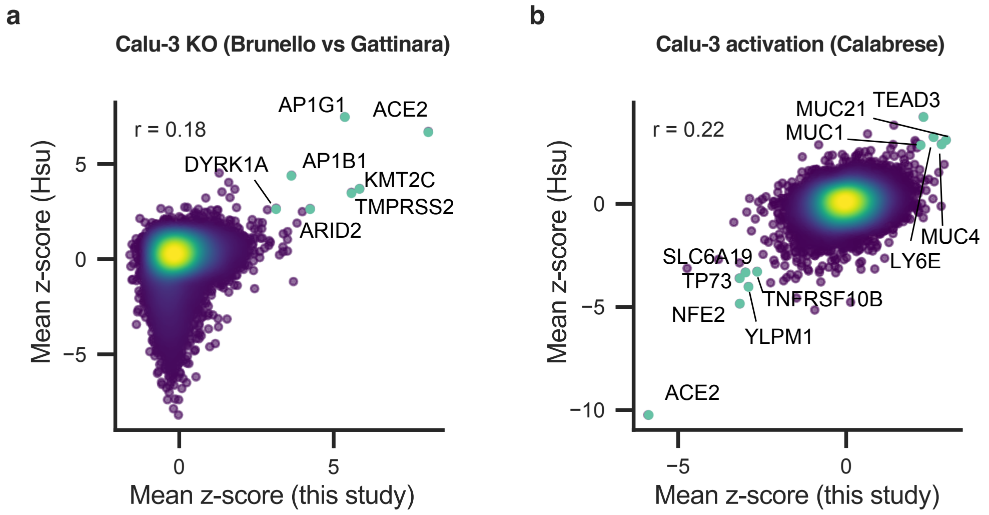

Fig. S7.
a. Comparison between this Calu-3 KO screen to the Calu-3 KO screen conducted by Hsu and colleagues [63]. Genes that scored among the top 20 resistance hits in both screens are annotated and shown in green.
b. Comparison between this Calu-3 activation screen to the Calu-3 activation screen conducted by Hsu and colleagues [63]. Genes that scored among the top 20 resistance hits and sensitization hits in both screens are annotated and shown in green.
